# Production and Functional Characterization of a Recombinant Predicted Pore-Forming Protein (TVSAPLIP12) of *Trichomonas vaginalis* in *Nicotiana benthamiana* Plants

**DOI:** 10.3389/fcimb.2020.581066

**Published:** 2020-09-30

**Authors:** Nicia Diaz, Chiara Lico, Cristina Capodicasa, Selene Baschieri, Daniele Dessì, Eugenio Benvenuto, Pier Luigi Fiori, Paola Rappelli

**Affiliations:** ^1^Department of Biomedical Sciences, University of Sassari, Sassari, Italy; ^2^Laboratory of Biotechnology, Italian National Agency for New Technologies, Energy and Sustainable Economic Development (ENEA) Casaccia Research Center, Rome, Italy; ^3^Mediterranean Center for Diseases Control, Sassari, Italy

**Keywords:** *Trichomonas vaginalis*, pore-forming protein, trichopore, recombinant protein *in planta*, microbial virulence

## Abstract

Pore-forming proteins (PFPs) are a group of functionally versatile molecules distributed in all domains of life, and several microbial pathogens notably use members of this class of proteins as cytotoxic effectors. Among pathogenic protists, *Entamoeba histolytica*, and *Naegleria fowleri* display a range of pore-forming toxins belonging to the Saposin-Like Proteins (Saplip) family: Amoebapores and Naegleriapores. Following the genome sequencing of *Trichomonas vaginalis*, we identified a gene family of 12 predicted saposin-like proteins (TvSaplips): this work focuses on investigating the potential role of TvSaplips as cytopathogenetic effectors. We provide evidence that *TvSaplip12* gene expression is potently upregulated upon *T. vaginalis* contact with target cells. We cloned and expressed recombinant TvSaplip12 *in planta* and we demonstrate haemolytic, cytotoxic, and bactericidal activities of rTvSaplip12 *in vitro*. Also, evidence for TvSaplip subcellular discrete distribution in cytoplasmic granules is presented. Altogether, our results highlight the importance of TvSaplip in *T. vaginalis* pathogenesis, depicting its involvement in the cytolytic and bactericidal activities during the infection process, leading to predation on host cells and resident vaginal microbiota for essential nutrients acquisition. This hence suggests a potential key role for TvSaplip12 in *T. vaginalis* pathogenesis as a candidate *Trichopore*.

## Introduction

*Trichomonas vaginalis* is a pathogenic protist responsible for human trichomoniasis, the most common non-viral sexually transmitted infection, with more than 156 million cases reported every year worldwide (Rowley et al., 2019). Infected women present a severe vaginitis usually associated to foul-smelling discharge, while in men infection is mostly asymptomatic (Kissinger, 2015). Trichomoniasis is associated with severe complications in pregnancy and with an increased susceptibility to Human Immunodeficiency Virus (HIV) infection and to cervical and prostate cancer (Twu et al., 2014; Fichorova et al., 2017; Han et al., 2019). During infection, the protozoon induces a cytopathic effect on the vaginal epithelium and on erythrocytes, which represent an important source of iron and fatty acids (Fiori et al., 1996).

Multiple mechanisms are involved in the colonization of the vaginal mucosa by *T. vaginalis*, with parasite adherence to target cells being a particularly relevant step in pathogenicity (Fiori et al., 1997; Hirt et al., 2007; Lustig et al., 2013). In fact, upon contact with host cells the parasite assumes an amoeboid shape, thus increasing the contact area and creating the ideal conditions for its cytolytic activity (Benchimol, 2004). The current view of trichomoniasis pathogenesis leading to host tissue damage involves on the one side the host inflammatory response, and on the other side the cytopathic effect exerted by the direct action of toxic proteins expressed by *T. vaginalis* (Mercer and Johnson, 2018). A number of proteins, mainly proteases, have been involved in the direct cytopathic effect mediated by *T. vaginalis* over epithelial target cells. In the past years we focused our efforts on the characterization of *T. vaginalis-*mediated heamolysis *in vitro*, showing a pore-forming activity leading to osmotic lysis of target cells in a pH, temperature, and Ca^2+^-dependent fashion (Fiori et al., 1993, 1996, 1997). Lysis of red blood cells (RBCs) by *T. vaginalis* occurs *in vivo*, where it is thought to be a mean of acquiring nutrients such as lipids and iron and may explain the observed exacerbation of symptoms during menses. Despite the detailed description of functional pore formation onto the membranes of target cells by *T. vaginalis*, the proteins putatively involved in this phenomenon have not been identified yet.

The ability to produce pore-forming proteins (PFPs) which mediates a cytolytic activity toward host cells and bacteria has been described also in other protozoan parasites, such as *Entamoeba histolytica, Trypanosoma cruzi*, and *Naegleria fowleri*. In particular, *E. histolytica* and *N. fowleri* PFPs, called Amoebapores and Naegleriapores, respectively, have been extensively characterized (Leippe, 2014). By sequence similarity, Amoebapores and Naegleriapores have been grouped into the family of saposin-like proteins (Saplip), a conserved protein family which can be found in many phylogenetically distant organisms (Bruhn, 2005). Although the members of the Saplip family perform many different biological functions, they are characterized by a common trait: the interaction with lipids. These proteins possess six conserved cysteine residues that are involved in forming the disulfide bond pattern characteristic of this protein family, along with a relative abundance of hydrophobic residues, resulting in a common folding (Hecht et al., 2004).

The bioinformatic analysis of *T. vaginalis* genome led to the identification of twelve putative genes (named *TvSaplip 1* to *12*) characterized by the presence of predicted saposin-like domains. *TvSaplip* genes (with the exception of *TvSaplip 9*) are transcribed in basal conditions (Carlton et al., 2007). The overall predicted aminoacidic sequence and structural homology of TvSaplip proteins to *Amoebapores* and *Naegleriapores* suggests a role as *trichopores* in the pathogenicity of *T. vaginalis* (Carlton et al., 2007).

Here we investigate the possible role of TvSaplips in *T. vaginalis* cytolytic activity. In order to identify *TvSaplip* genes involved in this process, we investigated their transcription upon contact of *T.vaginalis* on target cells. We showed that the *TvSaplip12* gene is upregulated upon contact with target cells and was therefore selected for further studies.

In order to characterize its possible role in pathogenicity, recombinant TvSaplip12 (rTvSaplip12) protein was produced *in planta*. Plant molecular farming is a well-established platform for the production of recombinant eukaryotic proteins (Sabalza et al., 2014), and several proteins of protozoan origin have been produced using plants as biofactories so far (Ghosh et al., 2002; Clemente et al., 2005; Chebolu and Daniell, 2007; Wang et al., 2008), but this list does not include any proteins from *T. vaginalis*. rTvSaplip12 protein was assayed for *in vitro* antibacterial, cytotoxic, and haemolytic activity. Finally, we also provide evidence for TvSaplip12 subcellular localization.

## Materials and Methods

### Microorganisms

*T. vaginalis* strain G3 was cultured by daily passages in trypticase-yeast extract-maltose (TYM) medium supplemented with 10% heat-inactivated bovine serum, 100 μg/ml streptomycin sulfate and 100 I.U./ml penicillin G, in 5% carbon dioxide atmosphere at 37°C (Diamond, 1957).

*Escherichia coli (ATCC25922)* and *Staphylococcus aureus (ATCC29213)* strains were grown in Luria-Bertani (LB) broth, at 37°C; *Agrobacterium tumefaciens* (GV3101 and C58C1) strains were grown in Yeast Extract Broth (YEB) medium, at 28°C. All bacterial strains were grown in constant agitation at 230 rpm.

### TvSaplip Genes Expression

Exponentially growing *T. vaginalis* (viability > 97%) were incubated at 37°C with RBCs (*T. vaginalis*/RBCs ratio 1:30) as described elsewhere (Fiori et al., 1996). After 0, 30, 60, and 90 min, cells were harvested and centrifuged at 350× g for 10 min. Total RNA was extracted from cell pellets using TRIzol reagent (Thermo-Fisher Scientific), according to manufacturer's instructions. Complementary DNA (cDNA) was obtained from 1 μg of total RNA using SuperScript IV Reverse Transcriptase and Random Hexamers (Invitrogen) as primers, following manufacturer's instructions.

Transcription of selected *TvSaplip* genes 1–8, 11, 12 (GenBank accession no: TVAG_388060, TVAG_209200, TVAG_393030, TVAG_473630, TVAG_000220, TVAG_453350, TVAG_070250, TVAG_213250, TVAG_306610, TVAG_183780) was evaluated by quantitative Reverse Transcription PCR (qRT-PCR). Specific TaqMan primer/probe sets have been designed by Beacon Designer Software (Premier Biosoft) for all *TvSaplip* genes except *TvSaplip7*. For *TvSaplip7* gene, due to its extremely small size, we developed a real-time PCR using the SYBR green as fluorescent chemical (Biorad, CA).

A conserved region of the 16S-like ribosomal gene of *T. vaginalis* was chosen as housekeeping gene (GenBank accession no: U17510.1). A series of multiplex qRT-PCR was designed by coupling each *TvSaplip* with the housekeeping gene. Primer/probe sequences and respective cycling conditions are described in Table 1.

**Table 1:** Gene ID, gene symbol, primers pair, probe sequence, and amplicon length of the selected genes.

**Gene ID**	**Gene symbol**	**Primers and probe sequence (5^**′**^ to 3^**′**^)**	**T.melting**	**Amplicon length**
(TVAG_388060) 1.665 bp	*TvSaplip1*	fw 5′-CGCCGAAATCTGCCAGAAGC-3′ rev 5′-TGACGCAGTAGTTGCATAATAGGC-3′ Probe 5′-CGCTGCCACAAAGGTTGCCCGCC-3′	59°C 68,8°C	89 bp
(TVAG_209200) 1.605 bp	*TvSaplip2*	fw 5′-TCTGCAAGAAGATCGGCCTTTG-3′ rev 5′-AATGGACAAGTTCAACGCACATG-3′ Probe 5′-TCCGTCAAGGGTGCCAAGTCCCAG-3′	58°C 65,9°C	90 bp
(TVAG_393030) 312 bp	*TvSaplip3*	fw 5′-TTCTTTGCATTATTGGGCCTCATG-3′ rev 5′-TTTCCGTCCTCAAGATAGTCTTCG-3′ Probe 5′-CTGCTGCTGCCGTTAAGCCACGT-3′	57,6°C 65,5°C	134 bp
(TVAG_473630) 1.149 bp	*TvSaplip4*	fw 5′-TTCTGTTCAACACAAGGAGTTACTG-3′ rev 5′-TCAATTGTTCCAACAACTTTTGTGC-3′ Probe 5′-CATGTCGCAGCCGCCTCCATTAACA-3′	57,2°C 65°C	77 bp
(TVAG_000220) 330 bp	*TvSaplip5*	fw 5′-ATCTACTTCCTTCGTTCTGGTG-3′ rev 5′-TTCTTGGTTTTCATAATATGCAGTG-3′ Probe 5′-AGCACCGATAAGTCTGCAAGCAGC-3′	55,5°C 64,7°C	90 bp
(TVAG_453350) 396 bp	*TvSaplip6*	fw 5′-CCAACCGAGATTTGCGAGAAGG-3′ rev 5′-CTCTGTTGAGAAAGCTGGTCTTCC-3′ Probe 5′-TGCCGCAAGGTTCGTGTTAACGCC-3′	58,8°C 66,1°C	123 bp
(TVAG_070250) 387 bp	*TvSaplip7*	fw 5′-TTAGGTAAATGCCGTCCAAAAG-3 rev 5′-TGAGAAATAATAAGGTGAATAAGA-3′	50°C	272 bp
(TVAG_213250) 381 bp	*TvSaplip8*	fw 5′-CTACAGCAAGCTCATGACATTCC-3′ rev 5′-TGTGGGAAGTGTGGAGCAAC-3′ Probe 5′-CGCGGCGGCCAGTCATCAACAGC-3′	58,4°C 69,8°C	147 bp
(TVAG_306610) 309 bp	*TvSaplip11*	fw 5′-GAAATCACAGAAAAGGTTGAAGC-3′ rev 5′-CTATGTACTTGATGATTTCTGGAAC-3′ Probe 5′-CGCATACCTTCAGGGTGATGCTCA-3′	55°C 63,7°C	109 bp
(TVAG_183780) 459 bp	*TvSaplip12*	fw 5′-CCGAAATATGTAAACTCTTTGATCC-3′ rev 5′-ATGGCAAAATTCTTGATTTGTTTTC-3′ Probe 5′-TCAGCCTGCATGGACTCGTTGAAC-3	55,5°C 64,3°C	122 bp
(Acc.n.U17510) 1.574 bp	*Trichomons vaginalis* *16s-like rRNA gene*	fw 5′-GGGAAACTTACCAGGACCAGATG-3′ rev 5′-AGCTGAATCAACGCTAGACAGG-3′ Probe 5′-CAACCCACGCACCACCAACGGC-3**′**	59°C 67,8°C	124 bp

qRT-PCR was performed in 25 μl of reaction containing 200 μM of each deoxyribonucleotide, 1.5 mM MgCl_2_, 300 nM of each primer and 200 nM hybridization probe, in PCR Buffer (Invitrogen, Italy), 1.25 U of Platinum Taq DNA polymerase (Invitrogen, Italy), and 3 μl of cDNA. All Multiplex qRT-PCR assays were run on an CFX96 Touch™ Real-Time PCR Detection System (Bio-Rad, CA) with the following cycling conditions: 40 cycles of denaturation at 95°C for 15 s, annealing for 15 s (see Table 1 for the annealing temperatures) and elongation at 72°C for 15 s.

*TvSaplip7-*specific qRT-PCR was performed using SYBR Select Master Mix (Applied Biosystems, US) according to the manufacturer's protocol, adding 300 nM of each primer, 3 μl of cDNA for each reaction and nuclease-free water (Invitrogen, Italy) to a total volume of 25 μl.

A standard curve was generated for each primer pair based on known quantities of cDNA. All calibration curves exhibited correlation coefficients higher than 0.99 and the corresponding qRT-PCR efficiencies were in the range 0.86–1.0. RNA without revertase treatment was used as negative control. All experiments were done in triplicate.

The relative change in gene expression of *TvSaplip 1* to *12* was analyzed employing the 2-ΔΔCt method (Livak and Schmittgen, 2001) using the Gene Expression Relative Quantitation Software Tool (Bio-Rad, CA) (available at http://www.gene-quantification.info/). Statistically significant expression changes were calculated using one-way ANOVA.

### Immunolocalization of TvSaplip12

*T. vaginalis* cells in logarithmic growth phase were washed and fixed with 4% paraformaldehyde in Phosphate Buffered Saline (PBS) pH 7.2, for 1 h. Cells were then washed three times in PBS, spotted on a slide and permeabilized with ice-cold methanol for 2 min. Slides were then incubated for 1 h with an anti-TvSaplip12 monoclonal antibody, obtained as previously described (Cuccuru et al., 2012).

After three washes in PBS-Tween 20 0.1%, slides were incubated with fluorescein isothiocyanate (FITC)-conjugated polyvalent anti-mouse antibody (Sigma-Aldrich) and 5 μg/ml 4′,6-diamidino-2-phenylindole (DAPI) for nucleus staining. An anti-*Salmonella abortus ovis* monoclonal antibody was used as negative control. Samples were observed using an Olympus BX51 microscope and the images were acquired with Optronics MagnaFire CCD Camera.

### Construction of the Plant Specific Expression Vector

A synthetic gene coding for a fusion of the Hemagglutinin (HA) (Niman et al., 1983) and 3xFLAG (Miceli et al., 1994; Einhauer and Jungbauer, 2001) peptides tags, the enterokinase cleavage site (for tags removal after protein purification), and the TvSaplip12 protein (GenBank accession no: TVAG_183780) was designed. The sequence was optimized referring to *N. benthamiana* codon usage, removing undesired cleavage sites and adding the *ClaI* and *SalI* restriction sites at the 5′ and the 3′ ends, respectively. The optimization process implied also the removal of possible deleterious sequence motifs such as putative intron splicing sites, CNG potential methylation sites, putative RNA branch point sequences, putative plant polyadenylation sites, potential termination sequences and RNA destabilizing sequences. Overall, the optimized sequence was obtained by modifying 50.5% of the native codons, but the AT percentage was unmodified. The TvSaplip12 sequence consists of 152 amino acids with a predicted molecular mass of 17.8 kDa, while the whole fusion protein consists of 188 amino acids with a predicted molecular weight of 22.1 kDa. The *in silico* optimized sequence was synthetized by a commercial supplier (Primm srl, Milano, Italy) and provided as a blunt ended PCR product. The purified 588 bp PCR fragment was initially inserted in a *SmaI*-digested pBlueScript-SK(+) cloning vector to perform sequence verification. After propagation in *E. coli* XL1Blue cells (Stratagene, Cedar Creek, TX), the *r(recombinant) TvSaplip12*-encoding sequence was *ClaI-SalI* moved in the properly digested plant expression binary vector pGR106 (Lu et al., 2003; kindly provided by Prof. David Baulcombe), generating the pGR106-Saplip plasmid. The pGR106 vector carries, as cDNA, the full genome of the plant virus Potato Virus X (PVX) with a cloning site inserted upstream the sequence encoding the viral coat protein. To assess the correct TvSaplip12 insertion, a PCR was performed on the pGR106-Saplip plasmid with primers that anneal up- and down-stream the TvSaplip12 encoding sequence (PVXFor 5′-CTGGGGAATCAATCACAGTGTTG-3′, PVXRev 5′-CAGTCTAGCTCTGCTGATGCCGTTG-3′), to be then used to transform *A. tumefaciens* GV3101 (pSOUP) cells through electroporation. Empty pGR106 vector was used as negative control.

### *Nicotiana benthamiana* Plants Vacuum Agroinfiltration

The rTvSaplip12 protein was transiently expressed in plant tissues by agroinfiltration (Sparkes et al., 2006). Briefly, *A. tumefaciens* GV3101 (pSOUP) cells carrying pGR106 or pGR106-Saplip plasmids were grown at 28°C in YEB medium, added with Rifampicin (50 mg/l), Tetracyclin (5 mg/l), and Kanamycin (50 mg/l), then centrifuged (5,000 g for 15 min) and the pellet resuspended in infiltration buffer [10 mM 2-(N-morpholino) ethanesulphonic acid, 10 mM MgCl_2_, pH 5.5]. The bacteria suspensions were infiltrated in leaves of *N. benthamiana* plants at 40 days from germination in a P2 greenhouse under standard controlled conditions: 16/8 h day/night cycle, 25°C, 84% humidity, daily light integral 3.9 moles/day (photosintetically active radiation 136 μmol/m^2^/s). The plants were submerged in a beaker containing the pGR106 or pGR106-Saplip bacterial suspension and 10-mmHg vacuum was then applied for 1 min in a vacuum bell jar (Sigma Chemical Co., MO). In this way, bacteria suspension was forced to penetrate into the intercellular space of mesophyll cells through the stomata. In the attempt to enhance transient expression efficiency, a group of plants was co-infiltrated with *A. tumefaciens* C58C1 (pCH32) cells carrying the binary vector p35S:p19, encoding the silencing suppressor protein P19 of Tomato Bushy Stunt Virus (TBSV) (P19-TBSV) (Danielson and Pezacki, 2013), testing different concentrations of bacteria (ranging from 0.4 to 1 O.D._600_). As further control, plants were infiltrated with buffer alone. All the leaves, except the apical ones (previously demonstrated to have a lower expression efficiency, Lombardi et al., 2009), were sampled at different days post infiltration (d.p.i), immediately weighted, frozen in liquid nitrogen and stored at −80°C.

### rTvSaplip12 Expression in Transiently Transformed Plants

To analyze separately the soluble protein fraction and proteins associated to membranes, leaves were homogenized (as described in Donald et al., 1993) 1:4 w/v in extraction buffer (GB, glycerol buffer: 400 mM sucrose, 100 mM TrisHCl pH 8.1, 10 mM KCl, 5 mM MgCl_2_, 10% glycerol, 10 mM β-mercaptoethanol) supplemented with a protease inhibitor cocktail (Complete, EDTA-free, Hoffmann-La Roche Ltd., Basel, Switzerland), and centrifuged for 30 min at 30,000 × g. This allows to obtain a high-speed pellet fraction, containing nuclei, chloroplast fragments, mitochondria and membranes derived from endoplasmic reticulum and disrupted organelles, while the supernatant is enriched in cytosolic and soluble proteins. Pellet fraction was resuspended in an equal volume of 1× Laemmli sample buffer, containing 8 M urea, boiled for 5 min and clarified by centrifugation at 15,000 × g for 5 min.

Proteins of soluble (20 μl) and membranes-associated (5 μl) fractions were separated on a 3–12% Tris-glycine gradient SDS-PAGE, in both reducing and non-reducing conditions, and submitted to Western Blot (WB) analysis. Briefly the resolved proteins were transferred to Polyvinylidene difluoride membrane (PVDF) (GE Healthcare Europe GmbH, Munich, Germany) using a Semi-Dry Transfer Unit (Hoefer TE70, GE Healthcare GmbH, Munich, Germany). After blocking in PBS containing 5% low fat milk (w/v) and washing in PBS-Tween 20 0.1%, anti-HA Horseradish Peroxidase (HRP)-conjugated (H-6533, Sigma Chemical Co., St. Louis) or anti-FLAG M2 HRP-conjugated (A-8592, Sigma Chemical Co., St. Louis, MO) antibodies, diluted 1:5,000 in 2% milk (w/v) in PBS, were used to detect rTvSaplip12. Conjugated antibodies were revealed by using Immobilon Western chemiluminescence HRP substrate (Millipore Merck).

### Quantification of Plant-Derived rTvSaplip12

To quantify rTvSaplip12 content in the soluble fraction, after total soluble protein quantification using a Bradford colorimetric assay (Bio-Rad, Hercules, CA), Enzyme Linked ImmunoSorbent Assay Maxisorp 96-well microtiter plates (NUNC) were coated in triplicate with 50 μg of proteins per well. After washing and blocking, the plates were incubated with the anti-FLAG M2 HRP-conjugated (A-8592, Sigma Chemical Co., MO) antibody, diluted 1:5,000 in 2% milk (w/v) in 1× PBS. Bound antibodies were revealed using 2,2′-azino-di-3-ethylbenz-thiazoline sulphonate (ABTS; KPL Inc., Gaithersburg, MD), and the colorimetric reaction measured with an ELISA reader at 405 nm. For the calibration curve a quantified purified protein fused to FLAG tag has been used.

### Antibacterial Activity of Plant-Derived rTvSaplip12

The antibacterial activity of rTvSaplip12 produced in *N. benthamiana* was tested on *S. aureus (ATCC29213)* and *E. coli (ATCC25922)* reference strains.

Briefly, 10 μl LB broth containing 10^7^ mid-logaritmic phase bacteria were added to 90 μl of total soluble protein extract of the plant agroinfiltrated with the pGR106-Saplip vector (containing ~450 ng of rTvSaplip12) for well, and 90 μl of the total soluble protein extract of a plant agroinfiltrated with the pGR106 empty vector, as negative control, in 96-wells plate. After 3 h and overnight incubation at 37°C, a total of 10 μl of bacterial suspension were harvested, diluted 1:10^5^ and 50 μl were plated in triplicate on LB-agar. After overnight incubation at 37°C, Colony Forming Units (CFU) were counted.

Collected data were analyzed and computed using Student *t*-test; a *P* ≤ 0.05 in one side testing was used as the criterion for statistical significance.

### Cytotoxic Activity of rTvSaplip12

Cytotoxicity of rTvSaplip12 was evaluated by treatment of *HeLa* cells grown in RPMI 1640 medium (Sigma Chemical Co.) supplemented with 10% inactivated fetal bovine serum, 100 I.U./ml penicillin, and 100 μg/ml streptomycin, at 37°C in a 5% CO_2_ atmosphere.

*HeLa* cells were seeded into a 96-well plate at the concentration of 3 × 10^4^ cells/well, then incubated for 12 h with plant extract containing different concentrations of rTvSaplip12 (0.3 μg/μl, 0.15 μg/μl, 0.075 μg/μl), obtained as described above. The extract of plants infiltrated with *A. tumefaciens* transformed with pGR106 empty vector was used as negative control.

Cell viability was evaluated by MTT (3-(4,5-dimethylthiazol-2-yl)-2,5-diphenyltetrazolium bromide) assay as described elsewhere (Mosmann, 1983). Percentages of growth inhibition, as 50% of the inhibitory concentrations (IC50) were calculated by linear regression analysis with 95% confidence limits. Each condition was tested in triplicate.

### Haemolytic Activity of rTvSaplip12

3 × 10^7^ human RBCs were added to 1 ml of PBS containing different concentrations of rTvSaplip12 plant extract (0.4 μg/μl, 0.2 μg/μl) obtained as described above, and incubated at 37°C. Samples were harvested at different time intervals and centrifuged at 350 × g. Hemoglobin release was quantified by spectrophotometric analysis of the supernatant, reading the absorbance at 546 nm. As negative and baseline control, RBCs were incubated in GB buffer alone; 100% of haemolysis was obtained incubating the same amount of RBCs in distilled water. RBCs incubated with the extract of plants agroinfiltrated with the pGR106 empty vector served as negative control.

## Results

### *TvSaplip* Genes Expression Upon Contact With Target Cells

In order to identify gene products potentially involved in *T. vaginalis-*induced haemolysis, the expression of *TvSaplip* genes upon contact with human RBCs was evaluated in qRT-PCR in a time-course experiment. This analysis was conducted on all *Tvsaplip* genes with the exception of *TvSaplip9* and −*10*, as the former is not actively transcribed (Carlton et al., 2007), while the latter was ruled out due to the presence in the *T. vaginalis* genome of two additional predicted ORFs (GenBank accession no. TVAG_392900, TVAG_114360, TVAG_518270) with an extremely high degree of homology with *TvSaplip10*, likely due to gene duplication events, and with the subsequent impossibility to test and characterize distinctively the gene products.

**Figure 1:**
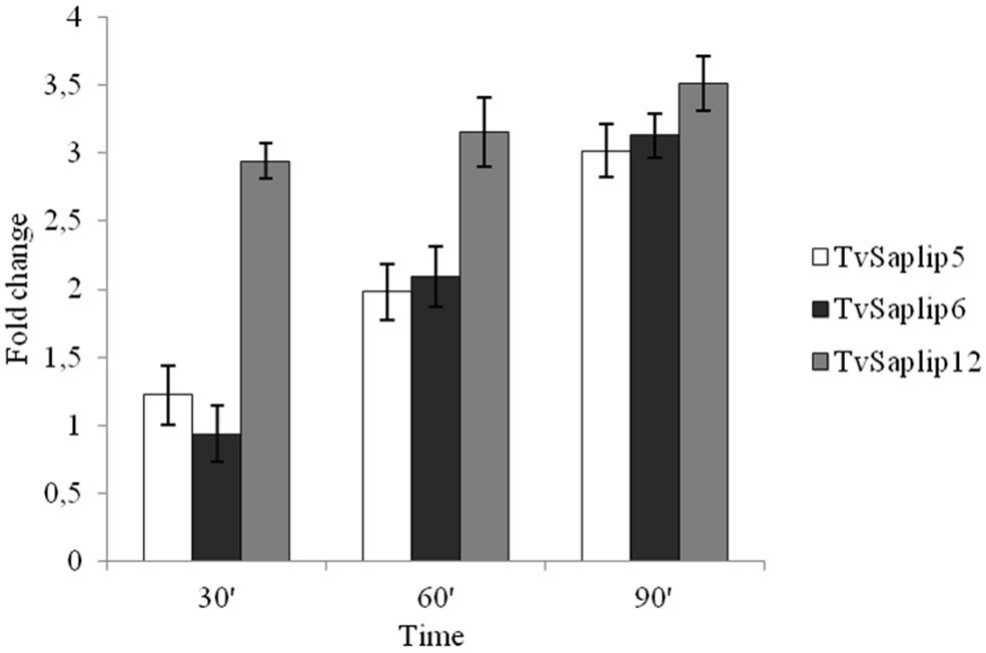
Relative expression analysis of *TvSaplip* genes. Relative expression analysis of *TvSaplip* genes in response to contact between *T. vaginalis* and RBCs after 30, 60, 90 min, evaluated by qRT-PCR. Bars represent relative expression levels averages ± SD from three independent experiments, with each condition tested in triplicate.

The qRT-PCR analysis highlighted that *TvSaplip1-4, and*−*7-8-11* transcription did not show any significant variation when compared to untreated controls (data not shown), whereas the transcription of *TvSaplip12* sharply increases of about 3.5-fold in the first 30 min of contact with target cells, and is maintained upregulated until the last time point (Figure 1). On the contrary, *TvSaplip5* and *6* show a 2-fold upregulation in transcription levels that occurs only after 60 min of incubation with RBCs. On the basis of these results *TvSaplip12* was selected for the subsequent characterization experiments.

### Cellular Localization of TvSaplip12

**Figure 2:**
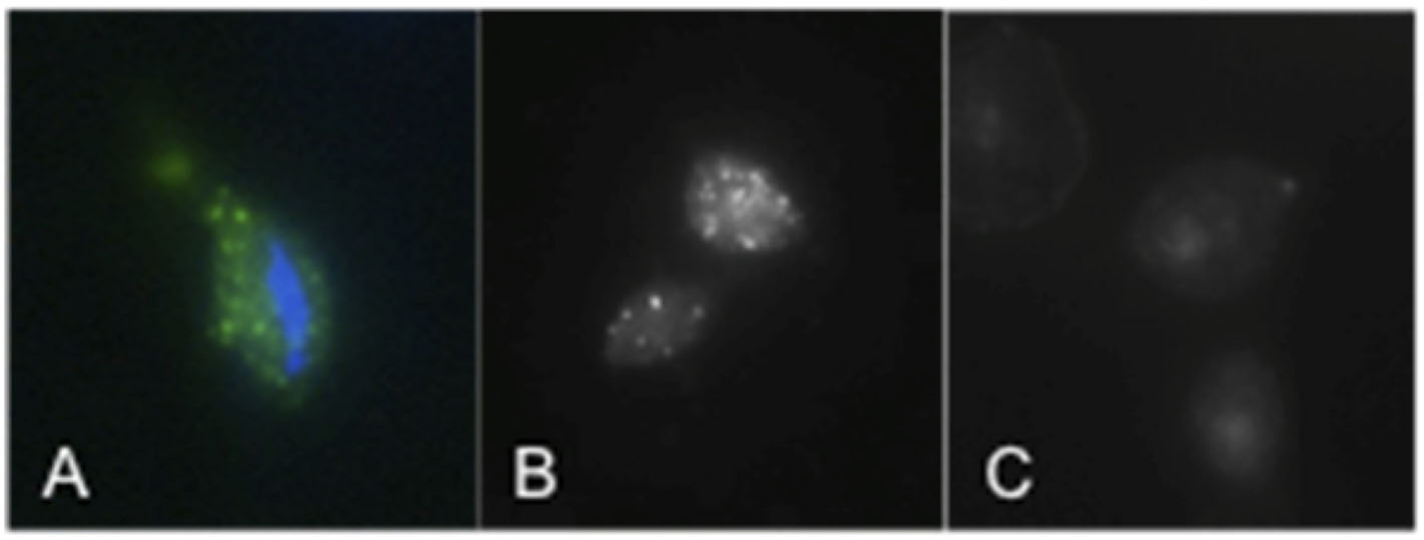
TvSaplip12 localization in *Trichomonas vaginalis* cells. **(A,B)** Tvsaplip12 was detected by immunofluorescence microscopy after staining with an anti-TvSaplip12 monoclonal antibody (green). Nuclei (blue) were stained with 4′,6′-diamidino-2-phenylindole (**A**: color image; **B**: black and white image). **(C)** No fluorescence was detectable using unrelated anti-*Salmonella abortus ovis* monoclonal antibody as negative controls.

Immunofluorescence assays using an anti-TvSaplip12 monoclonal antibody were performed to confirm that TvSaplip12 is expressed in *T. vaginalis* cells and to study its subcellular localization. In these experiments, anti-TvSaplip12 specifically stained numerous small granules uniformly distributed in the cytoplasm of *T. vaginalis* cells, demonstrating that the protein has a distinct focal distribution in the cytosol, possibly in intracellular storage vesicles (Figure 2).

### Production of Recombinant TvSaplip12

**Figure 3:**
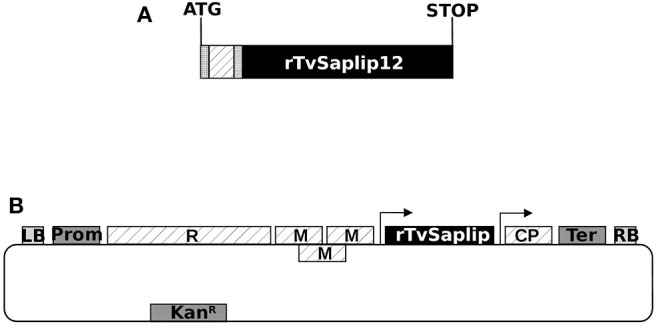
Scheme of the modified *TvSaplip12* sequence and of the pGR106-Saplip plant expression vector. **(A)** Diagram representing the *rTvSaplip12* gene. checked box: HA tag peptide encoding sequence; dotted box: 3xFlag tag peptide encoding sequence; striped box: enterokinase cleavage site. **(B)** Diagram of pGR106-Saplip plant expression vector. R, viral replicase; M, viral movement proteins; CP, viral coat protein; Prom, Cauliflower Mosaic Virus 35S Promoter; Ter, nopaline synthase gene terminator of *Agrobacterium tumefaciens* LB and RB, Left Border and Right Border. Arrows indicate the duplicated CP subgenomic promoter. KanR, neomycin phosphotransferase II.

A synthetic sequence optimized for the expression of TvSaplip12 in *N. benthamiana* plants was designed (Figure 3A), with tags useful for the purification through the Tandem Affinity method (Puig et al., 2001). The rTvSaplip12 was produced exploiting a transient expression strategy that couples the efficiency of Agrobacterium-mediated transformation with viral speed of expression through the plant expression vector pGR106 (Figure 3B).

**Figure 4:**
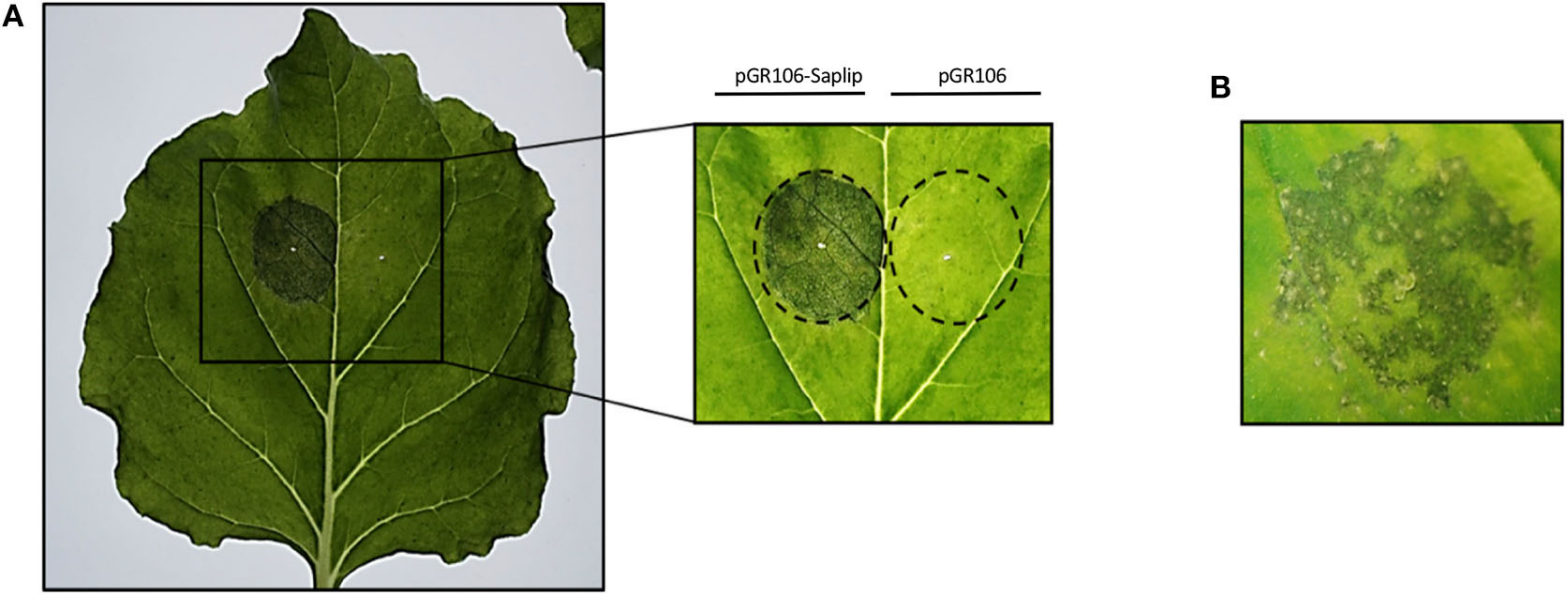
Effect of TvSaplip12 transient expression in plant tissue. **(A)** Typical phenotype of *N. benthamiana* leaves expressing TvSaplip12 at 6 d.p.i. Leaf was infiltrated with pGR106-Saplip-harboring agrobacteria (1 O.D.—left panel), and with the pGR106 empty vector as negative control (right panel). The inset shows more in detail the result. **(B)** A detail of a leaf agroinfiltrated with the pGR106-Saplip construct at 8 d.p.i., showing a necrotic spot symptomatology.

Based on preliminary experiments, the lowest concentration of agrobacteria was chosen to infiltrate plants for rTvSaplip12 production (0.4 O.D._600_ pGR106-Saplip plus 0.4 O.D._600_ p35S:p19), because higher bacterial concentrations (which usually result in higher proteins expression) caused a severe deterioration of the infiltrated plant tissues, may be related to the expression of the protein. In fact, as shown in Figure 4, plants infiltrated with bacteria harboring the pGR106-Saplip construct concentrated above 0,4 O.D._600_ showed at 6 d.p.i. severe symptoms (i.e., drying and maceration in the halo of infection; Figure 4A), and at 8 d.p.i. also the onset of single necrotic spots (Figure 4B), while no alterations were observed in plants infiltrated with agrobacteria harboring the pGR106 empty vector (Figure 4A).

The use of a lower concentration of agrobacteria resulted in a reduced deterioration of plant tissues, but also in a reduction of the expression levels that reached a maximum at 7 d.p.i. (data not shown).

**Figure 5:**
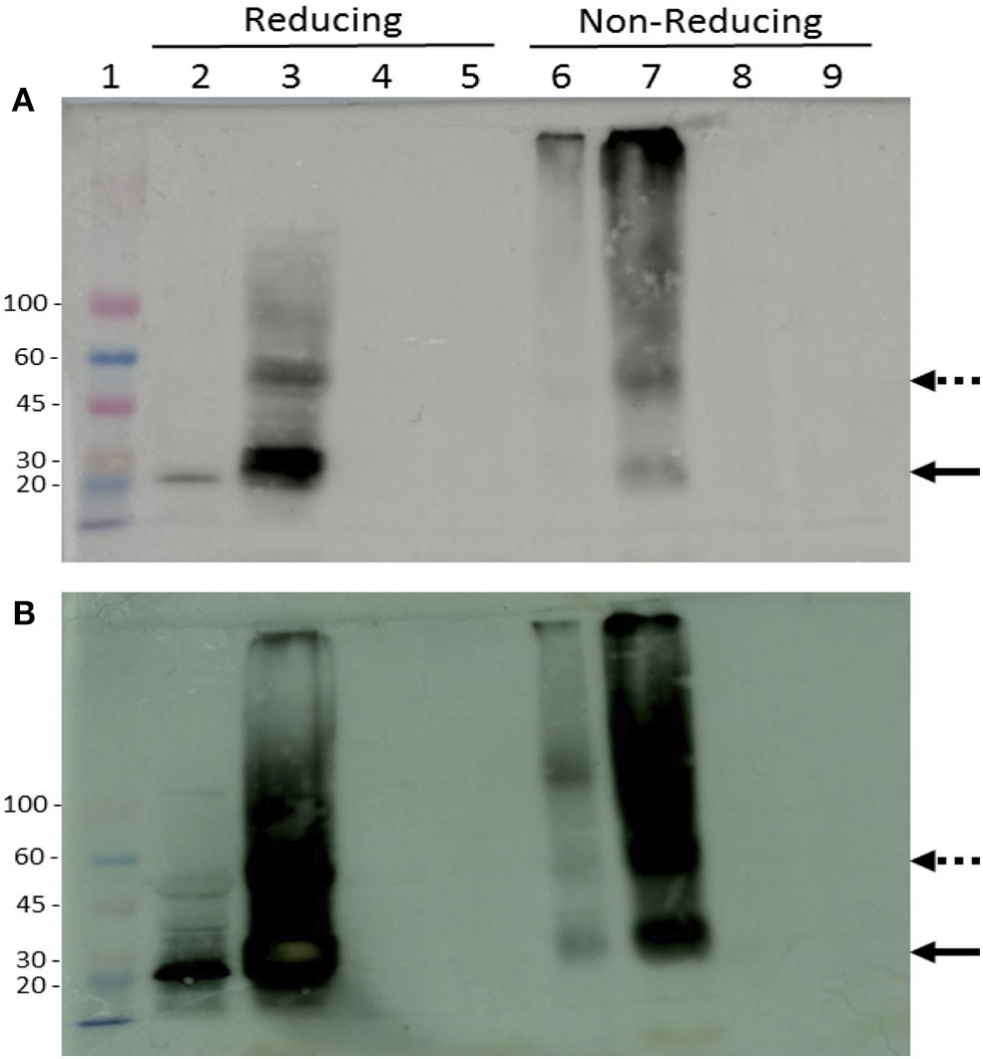
Immunodetection of tagged rTvSaplip. Western blot analysis of plant extracts on a 3–12% gradient SDS-PAGE with HRP-conjugated anti-HA **(A)** or anti-Flag **(B)** monoclonal antibodies. Samples were analyzed under both reducing and non-reducing conditions. Lane 1: Colorburst Molecular Weigth Marker (SIGMA Aldrich). Lanes 2 and 6: soluble cytosolic fraction of pGR106-Saplip infiltrated leaves; lanes 3 and 7: pellet membrane fraction of pGR106-Saplip infiltrated leaves; lanes 4 and 8: soluble cytosolic fraction of pGR106 empty vector infiltrated control leaves, lanes 5 and 9: pellet membrane fraction of pGR106 empty vector infiltrated control plant. Arrows indicate the monomeric (solid arrow) and dimeric (dotted arrow) forms of the TvSaplip12 protein.

The expression of rTvSaplip12 in *N. benthamiana* plants was verified by western blot analysis under reducing and non-reducing conditions by using antibodies directed against the HA and 3×FLAG tag peptides. Both total soluble proteins, and proteins associated to membranes and organelles (insoluble fraction) obtained from infiltrated plant tissues were analyzed. Under reducing conditions, a band corresponding to the expected molecular size (22 kDa) of rTvSaplip12 was detected in both samples (Figures 5A,B, lanes 2 and 3), although the signal was much more intense in the precipitated membrane-associate protein fraction. Under non-reducing conditions, the monomeric 22 kDa band was significantly less represented, and additional bands of higher molecular weight, probably representing dimers, multimers and/or aggregates of rTvSaplip12 (Figures 5A,B, lanes 6 and 7) were detected. The quantification of the protein content by ELISA indicated that rTvSaplip12 represented about 1.1% of the soluble proteins in the extracts.

Probably due to the rTvSaplip12 accumulation in the membrane fraction and to the low expression levels obtained for the cytosolic form, the attempt to purify the soluble monomeric form of the protein through affinity chromatography failed and protein activity was tested using the whole soluble fraction.

### Antibacterial Activity of rTvSaplip12

**Figure 6:**
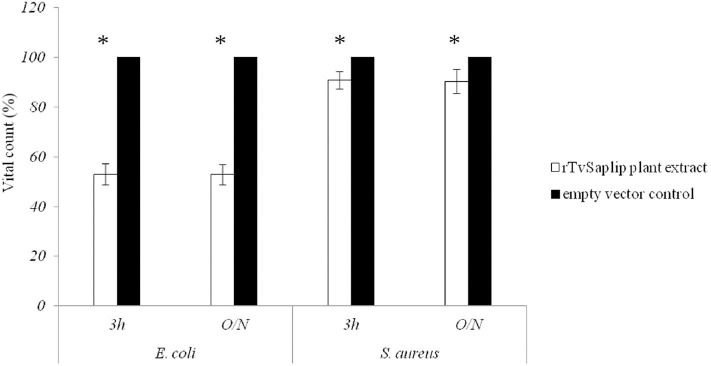
Antibacterial activity of rTvsaplip12. Bacterial CFU counts after treatment with rTvSaplip12 (white bars) and pGR106 empty vector (black bars) plant extracts (3 h and overnight, O/N). Bars represent means with standard deviation of three replicates and are representative of three independent experiments (*p < 0.05, unpaired Student *t*-test).

The antibacterial activity of plant-produced rTvSaplip12 was evaluated on *E. coli* and *S. aureus* cultures. The plant extract containing rTvSaplip12 showed significant antibacterial activity on *E. coli* after 3 h of incubation (44.6% reduction of CFU compared to controls). The effect does not increase significantly after an overnight incubation with a 47.12% reduction in the number of CFU. On the contrary, the effect observed on *S. aureus* did not appear to be relevant, with a minimal reduction in CFU count both after 3 h or overnight incubation (respectively, 5.6 and 9.7%) (Figure 6). Control plant extract not containing rTvSaplip12 did not show any antibacterial activity against either bacterial strain.

### Cytotoxic and Haemolytic Activity of rTvSaplip12

The cytotoxic effect of rTvSaplip12 was evaluated by incubating overnight *HeLa* cells with the plant extract containing the recombinant protein. An extract of plants agroinfiltrated with the pGR106 empty vector has been used as negative control.

**Figure 7:**
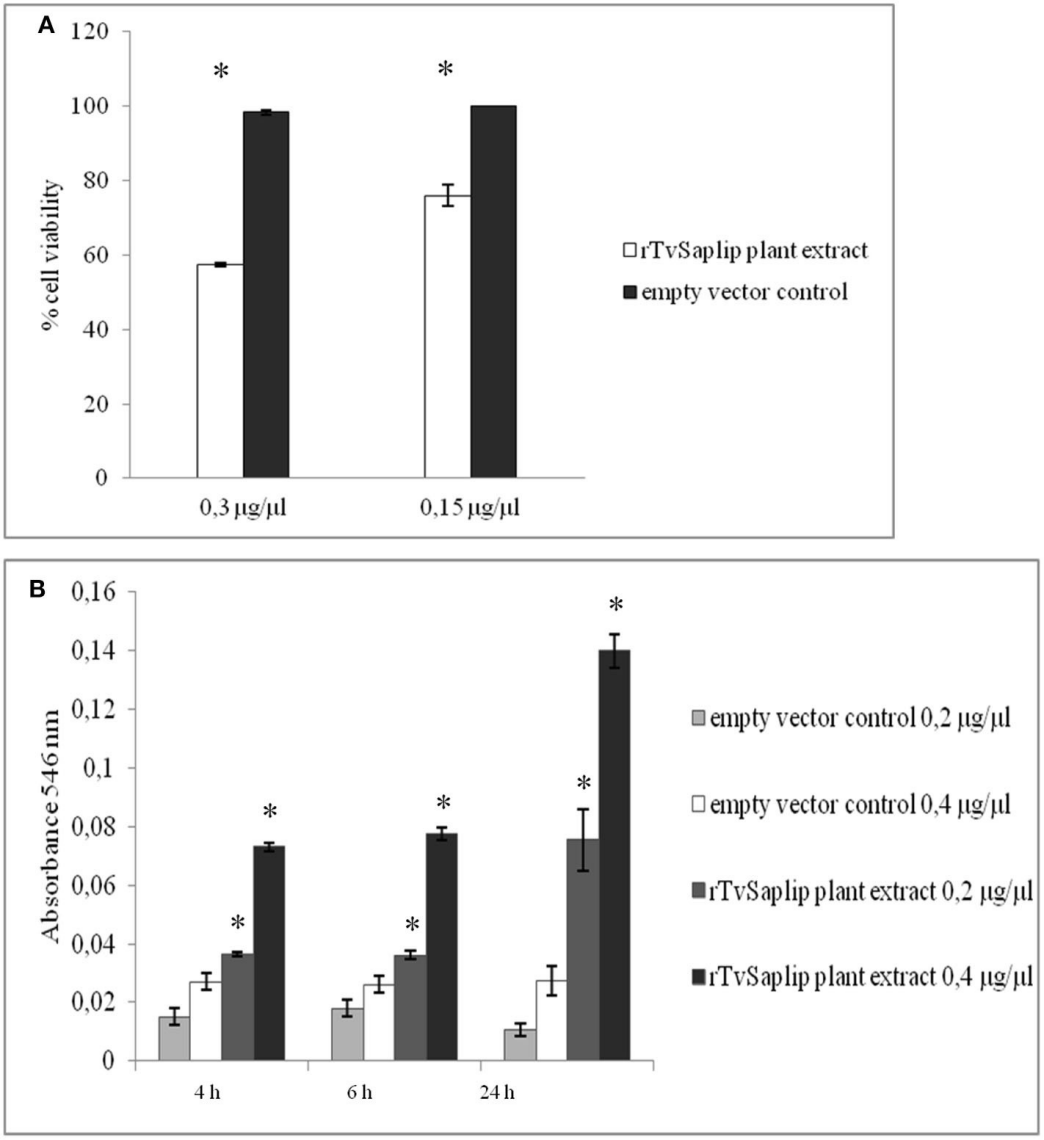
Cytotoxic and haemolytic activity of rTvSaplip12. **(A)** Cytotoxic activity of different concentrations of rTvSaplip12 and pGR106 empty vector plant extracts was quantified by monitoring cell viability using MTT method after 24 h of incubation. Bars represent means with standard deviation of 3 replicas and are representative of tree independent experiments (*p < 0.05, unpaired Student *t*-test). **(B)** Haemolytic effect of different concentrations of rTvSaplip12 and pGR106 empty vector plant extracts, assessed via monitoring hemoglobin release at different time points. Bars represent means with standard deviation of 3 replicates and are representative of three independent experiments (*p < 0.05, unpaired Student *t*-test).

*HeLa* cells were susceptible to lysis induced by rTvSaplip12 in a dose-dependent manner, with 0.3 μg/μl of the recombinant protein determining a reduction in cell viability of about 43%, as compared to controls (Figure 7A).

Similarly, rTvSaplip12-containing plant extract showed a significant haemolytic activity on human RBCs at both concentrations tested, while the same concentrations of the plant extract alone did not show any haemolytic effect. Figure 7B shows a time- and dose-dependency of the haemolytic effect of rTvSaplip12-containing plant extracts.

## Discussion

*T. vaginalis* is able to induce host cell death *in vitro* in a plethora of target cells, ranging from vaginal epithelial cells and prostate cells (Lustig et al., 2013; Lin et al., 2015) to B and T lymphocytes, via different mechanisms involving pyroptosis (Riestra et al., 2019) and protease-mediated apoptosis (Sommer et al., 2005; Quan et al., 2014), among others. A peculiar physiological target cell of *T. vaginalis* is represented by human RBCs. *T. vaginalis* displays a temperature, pH, Ca^2+^, contact-dependent haemolytic activity *in vitro*, involving the functional formation of pores on RBCs membranes (Fiori et al., 1999) and thus suggesting the production by the protist of pore-forming proteins (PFP) with cytotoxic activity. Despite the detailed characterization of the haemolysis induced by *T. vaginalis*, the proteins mediating such activity have never been identified.

The ability to produce a pore-forming activity is a trait common to other pathogenic protozoa, e.g., *E. histolytica, T. cruzi*, and *N. fowleri*, whose PFPs have been extensively characterized. Amobeapores and Naegleriapores, the PFPs of *E. histolytica* and *N. fowleri*, belong to a protein family known as saposin-like proteins (Saplip), characterized by a conserved domain showing a specific six-cysteine pattern and a relative abundance of hydrophobic residues.

Here we investigate the contribution of TvSaplip12, a homolog of Amoebapores and Naegleriapores, to *T. vaginalis*-mediated cytotoxic and bacteriolytic activity. The *TvSaplip12* gene is a component of a gene family of 12 members (*Tvsaplip1-12* previously identified by our research group in a genomic survey; Carlton et al., 2007), here selected as subject for functional analysis following qRT-PCR experiments aimed at assessing the transcription regulation of the *TvSaplip* family. We showed that upon contact with RBCs the transcription of *TvSaplip12* rapidly and steadily undergoes a 3.5-fold upregulation, in a markedly distinct manner from other *TvSaplip* genes.

To investigate the activity of the corresponding protein, TvSaplip12 was expressed in a recombinant form using a well-characterized low-cost plant expression system, suitable for the production of a wide spectrum of recombinant proteins. “Plant Molecular farming,” by consolidating its performances brought on the market several products, not only for diagnostic or industrial applications (Hood, 2002) but also for the treatment of human diseases (Zimran et al., 2011). To date, proteins from different pathogens (e.g., virus and bacteria) have been successfully produced in different plant hosts, mainly for vaccine development purposes. Also several protozoan proteins have been expressed in plants (Daniell et al., 2009), such as the surface proteins pf38 and pfMSP1 19 of *Plasmodium falciparum* (Ghosh et al., 2002; Feller et al., 2013), the Gal/GalNAc lectin of *Entamoeba histolytica* (Chebolu and Daniell, 2007), the surface protein 4/5 of *Plasmodium yoelii* (Wang et al., 2008), and the SAG1 and GRA4 antigens of *Toxoplasma gondii* (Clemente et al., 2005; Yácono et al., 2012).

Over time, highly efficient strategies have been set up to optimize expression levels in plants. In the present paper, a strategy based on the use of *A. tumefaciens* as vehicle of an expression vector based on the plant virus PVX was adopted.

Plant tissues infiltrated with the bacteria harboring the TvSaplip12 construct successfully expressed the recombinant protein. Nonetheless, plants showed severe alterations six d.p.i. The detrimental effects visible on plant leaves could be ascribed to the expression of the recombinant protein, since they are not present in the controls, suggesting that a pore-forming/toxic activity may be maintained also in plants. This hypothesis is also supported by the observation that the protein accumulates mainly in multimeric or aggregated forms in the membranes containing fraction, as described for other members of the Saplip protein family (Herbst et al., 2002). This association to membranes unfortunately sequestered most of the expressed protein from the soluble fraction, leading to an underestimation of its expression and hampering its purification from plant extracts.

Since the sequence of TvSaplip12 is highly similar to those of Amoebapores and Naegleriapores proteins, which are characterized by a well-documented antibacterial and cytolytic activity (Herbst et al., 2002, 2004; Leippe and Herbst, 2004; Michalek et al., 2013) we analyzed the effect of rTvSaplip12 on bacterial and human cells. In our hands, plant extracts containing rTvSaplip12 show a remarkable antibacterial activity against *E. coli*, while *S. aureus* cultures were not significantly affected. It is indeed known that, because of the rigidity of the cell wall, Gram-positive bacteria are able to withstand turgor pressures 3 to 25 times higher those tolerated by Gram-negative bacteria (Koch and Pinette, 1987) and this, together with other features of cell wall composition, may be one of the reasons why Gram-positive bacteria are typically more resistant to antimicrobial peptides (Sato and Feix, 2006). This observation is consistent with that obtained using the antimicrobial peptide Cecropin B (Moore et al., 1996).

In order to establish a potential role as *Trichopore* for TvSaplip12, we set up experiments to assess the cytotoxic activity of the recombinant protein. *HeLa* cells challenged with rTvSaplip12-containing plant extracts showed a 43% reduction in viability, while in haemolysis assays RBCs similarly showed to be subjected to cell death after rTvSaplip treatment. Despite we could not rule out *a priori* that the cytotoxic activity might hypothetically be achieved through spontaneous interaction of rTvSaplip12 with other plant proteins present in the extract, altogether our results are strongly suggestive of a central role for this protein in *T. vaginalis*-induced target cell death. Importantly, our data show that TvSaplip12 also has the potential for being depicted as a key molecule in nutrient acquisition during infection: indeed, *T. vaginalis* predates on resident bacteria and targets RBCs as a means to scavenge essential molecules such as iron and precursors for macromolecular biosynthesis.

In our experiments we also demonstrated that TvSaplip12 is compartmentalized in discrete cytoplasmic granules in the *T.vaginalis* cell, indicating that the protein may be released by exocytosis mechanisms, as previously demonstrated for Amoebapores (Andrä et al., 2003). This cellular localisation also suggests a possible role of TvSaplip12 in bacterial lysis after phagocytosis.

Altogether, our results are consistent with the hypothesis that the protein encoded by the *TvSaplip12* gene can be described as a PFP, and that this protein may be involved in the cytolytic activity of *T. vaginalis* during the infection process. The partial functional characterization of the protein was performed through plants as eukaryotic protein expression system: TvSaplip12 is the first *T. vaginalis* protein expressed by using “Plant Molecular farming.” Further studies are needed to confirm the prominent role of TvSaplip12 in *T. vaginalis* pathogenetic effect, and to assess the specific mechanisms by which the cytolytic and bactericidal activity is accomplished. The use of recently developed genetic tools for *T. vaginalis* such as CRISPR/Cas9 (Janssen et al., 2018) will make gene modification and gene knock-out approaches, which will potentially allow to dissect the specific functions and roles of this candidate *Trichopore*, possible. Further work would also allow to elucidate the functions of the other putative *TvSaplip* gene products identified in the *T. vaginalis* genome sequence, contributing to shed light on the complex series of events taking place at the host:pathogen interface.

Indeed, a more complex picture of the molecular actors involved in the cytolytic mechanism exerted by *T. vaginalis* can support the development of effective drugs and vaccines.

## Data Availability Statement

All datasets generated for this study are included in the article/supplementary material.

## Author Contributions

ND and CL performed experiments and prepared figures and table. CC, SB, and DD critically revised the manuscript and analyzed the data. EB critically revised the manuscript. PF and PR designed the study, contributed to interpretation of results, and planned the experiments. All authors wrote the paper.

## Conflict of Interest

The authors declare that the research was conducted in the absence of any commercial or financial relationships that could be construed as a potential conflict of interest.
